# The Expression of Virulence Factors in *Vibrio anguillarum* Is Dually Regulated by Iron Levels and Temperature

**DOI:** 10.3389/fmicb.2019.02335

**Published:** 2019-10-15

**Authors:** Marta A. Lages, Miguel Balado, Manuel L. Lemos

**Affiliations:** Department of Microbiology and Parasitology, Institute of Aquaculture, Universidade de Santiago de Compostela, Santiago de Compostela, Spain

**Keywords:** *Vibrio anguillarum*, fish pathogens, RNA-Seq, transcriptome, virulence factors, piscibactin, vanchrobactin

## Abstract

*Vibrio anguillarum* causes a hemorrhagic septicemia that affects cold- and warm-water adapted fish species. The main goal of this work was to determine the temperature-dependent changes in the virulence factors that could explain the virulence properties of *V. anguillarum* for fish cultivated at different temperatures. We have found that although the optimal growth temperature is around 25°C, the degree of virulence of *V. anguillarum* RV22 is higher at 15°C. To explain this result, an RNA-Seq analysis was performed to compare the whole transcriptome profile of *V. anguillarum* RV22 cultured under low-iron availability at either 25 or 15°C, which would mimic the conditions that *V. anguillarum* finds during colonization of fish cultivated at warm- or cold-water temperatures. The comparative analysis of transcriptomes at high- and low-iron conditions showed profound metabolic adaptations to grow under low iron. These changes were characterized by a down-regulation of the energetic metabolism and the induction of virulence-related factors like biosynthesis of LPS, production of hemolysins and lysozyme, membrane transport, heme uptake, or production of siderophores. However, the expression pattern of virulence factors under iron limitation showed interesting differences at warm and cold temperatures. Chemotaxis, motility, as well as the T6SS1 genes are expressed at higher levels at 25°C than at 15°C. By contrast, hemolysin RTX pore-forming toxin, T6SS2, and the genes associated with exopolysaccharides synthesis were preferentially expressed at 15°C. Notably, at this temperature, the siderophore piscibactin system was strongly up-regulated. In contrast, at 25°C, piscibactin genes were down-regulated and the vanchrobactin siderophore system seems to supply all the necessary iron to the cell. The results showed that *V. anguillarum* adjusts the expression of virulence factors responding to two environmental signals, iron levels and temperature. Thus, the relative relevance of each virulence factor for each fish species could vary depending on the water temperature. The results give clues about the physiological adaptations that allow *V. anguillarum* to cause infections in different fishes and could be relevant for vaccine development against fish vibriosis.

## Introduction

*Vibrio anguillarum* is a marine bacterium inhabitant of the estuarine and marine coastal ecosystems worldwide. It is an important fish pathogen since it is the etiological agent of classical vibriosis in warm- and cold-water fish species leading to high mortalities and economic losses in aquaculture (Toranzo et al., 2017). Bivalve molluscs and crustaceans are also occasionally affected by this bacterium (Aguirre-Guzman et al., 2004; Paillard et al., 2004). *V. anguillarum* isolates are classified into 23 different O-serogroups (O1 to O23) (Pedersen et al., 1999), the most virulent strains belong to serotypes O1, O2, and, to a lesser extent, O3. The remaining serotypes are mostly environmental strains isolated from seawater, marine animals, or sediments (Frans et al., 2011; Toranzo et al., 2017).

As a marine bacterium, *V. anguillarum* must be able to adapt its physiology to environmental fluctuations in salinity, availability of nutrients or temperature. It is an eurythermal bacterium capable of growing in a wide range of temperatures, from 10 to 35–42°C, with its optimal growth temperature around 25–30°C (Guérin-Faublée et al., 1995). Seasonal rising of seawater temperature has been associated with the proliferation of *Vibrio* species (Maeda et al., 2003), and hence, the occurrence of some fish diseases caused by these bacteria is also increased (Le Roux et al., 2015). *V. anguillarum* can cause vibriosis at low temperatures (5–18°C) (Ma et al., 2017), with 15°C considered as the optimal temperature to cause vibriosis outbreaks (Austin and Austin, 2007; Bellos et al., 2015). Thus, vibriosis episodes, as most ectothermic aquatic animal infectious diseases (Guijarro et al., 2015), usually occur at a temperature lower than that for the optimal growth of the bacteria. In addition, psychrotrophic *V. anguillarum* strains were also isolated from farmed fish in Norway causing diseases at water temperatures of 1–4°C (Olafsen et al., 1981). Remarkably, *V. anguillarum* survives in a cultivable form for more than a year in saline water (Hoff, 1989) where darkness, coldness (around 5°C), and anaerobiosis are environmental factors that help the pathogen to survive (Eguchi et al., 2000). This favors the dissemination and presence of *V. anguillarum* in seawater and sediments in all seasons (Muroga et al., 1986; Maeda et al., 2003).

The mechanisms of *V. anguillarum* that cause disease in fish are not yet completely understood; nonetheless, a considerable number of virulence factors have been identified. They include motility, chemotaxis, LPS, extracellular products with hemolytic and proteolytic activities, and multiple iron-uptake systems (Li and Ma, 2017; Toranzo et al., 2017). Exopolysaccharide production is required for biofilm formation and colonization of skin mucosal layer in the initial stages of infection (Croxatto et al., 2007; Weber et al., 2010). To date, the most relevant virulence factors identified in *V. anguillarum* are the metalloprotease EmpA (Crisafi et al., 2014), hemolysins such as Vah1 (Hirono et al., 1996), Vah2–5 or Rtx (Rodkhum et al., 2006; Li et al., 2008), and a complete set of iron uptake mechanisms including genes for heme utilization (Mouriño et al., 2004), transport of unchelated ferrous iron (*feoABC*) and, most notably, three siderophore systems vanchrobactin, anguibactin, and piscibactin (Li and Ma, 2017; Balado et al., 2018). Interestingly, we recently found that many highly pathogenic *V. anguillarum* strains are able to produce two siderophores simultaneously: vanchrobactin and piscibactin (Balado et al., 2018). Since piscibactin has a dual requirement of iron starvation and low temperature to be synthesized, the production of each siderophore is balanced in a temperature-dependent manner. It was postulated that piscibactin production would enhance niche flexibility conferring wide virulence properties even at low temperatures (Balado et al., 2018). In addition, some recent studies have demonstrated that environmental stress could affect the expression of virulence genes that are involved in the ability of *V. anguillarum* to cause infection (Crisafi et al., 2014; Guanhua et al., 2018).

The ability of a bacterial pathogen to cause disease depends not only on the presence of virulence factors but also, and more importantly, on a tight control of their expression. The main goal of this work was to determine the temperature-dependent changes in the transcriptome that could identify virulence properties of *V. anguillarum* for fish cultivated at cold- or warm-water temperatures. For this purpose, an RNA-Seq analysis was performed to compare the whole transcriptome profile of *V. anguillarum* strain RV22 cultured under low-iron availability at either 25 or 15°C, which would mimic the conditions that *V. anguillarum* finds during host colonization at warm- or cold-water temperatures.

## Materials and Methods

### Bacterial Strains and Culture Media

We have used *V. anguillarum* strain RV22 (serotype O2) to study the temperature-dependent virulence properties of *V. anguillarum* since it is closely related to HI610, a strain that was reported to cause high mortality ratios at temperatures below the optimal growth (Rønneseth et al., 2017). Both strains share 97.6782% of their genome sequence with an identity of 99.9689% according to data from NCBI genome pairwise comparison (accession no.: HI610, GCA_001989835.1; RV22, GCA_000257185.1).

*Vibrio anguillarum* RV22 was routinely grown at 25°C on tryptic soy agar (TSA) or broth (TSB) (Cultimed) supplemented with 1% NaCl (TSA-1 or TSB-1). Stock cultures were kept frozen at −80°C in TSB-1 with 15% (v/v) glycerol. CM9 minimal medium (Miller, 1992) was also used in some assays.

### Fish Virulence Assays

The virulence assay was carried out with Senegalese sole (*Solea senegalensis*) fingerlings with an average weight of 10 g. Fish were divided into four groups of 30 animals. Two fish groups were maintained in 50-L seawater tanks at 15°C and the others at 25°C with continuous aeration. Fish were inoculated intraperitoneally (ip) with 0.1 ml of bacterial cell suspension of *V. anguillarum* RV22. The dose used was 2–5 × 10^5^ CFU per fish obtained by 10-fold serial dilutions of a bacterial suspension at an OD_600_ = 0.5 prepared by resuspending several colonies from a 24-h TSA-1 culture into saline solution (0.85% NaCl). The precise number of injected bacterial cells was determined by plate count of 10-fold serial dilutions on TSA-1. Two control groups (one per each temperature) were inoculated with 0.1 ml of saline solution (0.85% NaCl) and maintained at 15 or 25°C. Mortalities were recorded daily for 10 days after injection and statistical significance of differences in survival curves was determined using the Kaplan–Meier method with Mantel–Cox log-rank test using SPSS (version 20; IBM SPSS Inc., Chicago, IL). *P* values were considered significant when *P* < 0.05. The protocol for animal experimentation used in this study followed the Spanish and European Union legislation and has been reviewed and approved by the Bioethics Committee of the University of Santiago de Compostela.

### Growth Conditions and Total RNA Extraction

*Vibrio anguillarum* RV22 was grown aerobically in CM9 minimal medium under iron-deficient (2,2′-dipyridyl 50 μM) conditions at two different temperatures: 25 and 15°C. These culture conditions would mimic those that the pathogen encounters during the infection of warm- and cold-water fishes. As reference controls, *V. anguillarum* was grown in CM9 supplemented with iron (Fe_2_SO_4_ 10 μM) at the same temperatures. Bacterial cells were harvested at the mid-log phase (OD_600_ ∼ 0.8) by centrifugation at 10,000 × *g* for 10 min and total RNA was isolated with RNAwiz (Ambion) following the manufacturer’s recommendations. RNA was isolated from three independent samples at each experimental condition (biological replicates). The quality and concentration of RNA were determined by 1% agarose gel visualization and by using the RNA 6000 NanoKit on the Bioanalyzer 2100 (Agilent Technologies, Palo Alto, CA). RNA samples were stored at −80°C until further utilization.

### cDNA Library Construction and Sequencing

RNA extraction from triplicate biological samples was used to construct independent cDNA libraries for whole-transcriptome sequencing. Elimination of residual DNA and bacterial rRNA depletion was done before library construction. Each biological replicate was represented in an independent library that consisted of ca. 20 M of 2 × 150 bp reads. Massive sequencing was performed in an Illumina MiSeq sequencing machine using NextSeq High Output 1 × 150 pb kit. Construction of cDNA Libraries and Illumina Sequencing was carried out by FISABIO Sequencing and Bioinformatics Service (Valencia, Spain). RNAseq reads were deposited at NCBI Sequence Read Archive (SRA) under accession no. SRP213600. Reads mapped statistics and accession numbers of different samples are shown in Supplementary Table S1.

### Bioinformatic Analysis and Gene Expression Quantification

RNA-Seq data analysis and validation were performed using the set of open source software programs included in Tuxedo suite (Ghosh and Chan, 2016). Briefly, reads were aligned to chromosomes I and II of *V. anguillarum* RV22 strain (GenBank accession no. GCA_000257185.1) using Tophat. Then, Cufflinks was used to assemble these mapped reads into possible transcripts and generate a final transcriptome assembly. Cuffdiff was used to identify differentially expressed genes and transcripts between each biological condition. The complete data set is shown in Supplementary Table S2. Finally, the R package CummeRbund was used to process the output files of Cuffdiff, resulting in an output in the form of quality plots and figures. Blast2GO was used for functional annotation, enzyme code mapping, and analysis of pathway maps. COG database and KEGG were used to DEGs functional classification. Subcellullar localization prediction was done using PSORTb v3.0 (Yu et al., 2010).

### Phenotypic Characterization

In order to validate some of the results obtained through RNA-Seq analysis, some phenotypic tests were evaluated under the same conditions used for RNA-Seq. We chose three traits that have been reported to be involved in the virulence of *V. anguillarum*: motility, formation of biofilm, and hemolytic activity.

Motility was measured by the soft agar method. An inoculum in CM9 at mid-log phase (OD_600_ ∼ 0.8) was stabbed into CM9 soft agar (CM9 supplemented with 0.4% agar) containing either Fe_2_SO_4_ 10 μM or 2,2′-dipyridyl 50 μM. Motility was evaluated by observing cloudiness after incubation at 15 or 25°C during 48 h. The test was repeated three times. *Photobacterium damselae* subsp. *piscicida* DI21 was used as a non-motile control.

Biofilm formation was evaluated by the crystal violet staining assay. Glass tubes containing 10 ml of CM9 containing either Fe_2_SO_4_ 10 μM or 2,2′-dipyridyl 50 μM were incubated at 25 or 15°C until mid-log phase (OD_600_ ∼ 0.8). The content of each tube was gently removed, and the remaining attached bacteria were fixed with 10 ml of methanol 99% (Panreac). The tubes were then stained for 5 min with 2 ml of crystal violet 2% (bioMérieux). Excess stain was rinsed off by placing the tubes under running tap water. After washing with PBS and air drying, the dye bound to the biofilm was solubilized with 10 ml of glacial acetic acid 33% (v/v) (Panreac). The absorbance was measured at 570 nm in a spectrophotometer (Hitachi U-2000). The assay was repeated three times. Statistical significance was determined by Student’s *t* test with a threshold *p* value < 0.05.

Hemolytic activity was detected in Columbia Agar plates (Oxoid). *V. anguillarum* RV22 was cultured in CM9 plates containing 2,2′-dipyridyl 50 μM at either 25 or 15°C for 24 and 48 h, respectively. A loopful of cell biomass, scratched from the surface of CM9 plates, were deposited on the surface of Columbia Agar plates. Hemolytic halos were inspected after 48 h of incubation at 25 or 15°C.

### β-Galactosidase Assay

The *V. anguillarum* RV22 strain carrying either plasmid pMB276 (P*frpA*:*lacZ* in pHRP309) or plasmid pMB277 (P*araC1*:*lacZ*) (Balado et al., 2018) was grown in CM9 minimal medium under iron starvation (2,2′-dipyridyl 50 μM) at 10, 15, or 25°C up to OD_600_ ∼ 0.3–0.4. Then, β-galactosidase activities were measured by the method of Miller (1992) as previously described (Balado et al., 2018). The results shown are the means of three independent experiments, with each measure being performed in triplicate. LacZ fusion of housekeeping gene *proC* (pML263) (Balado et al., 2018) was used as constitutive control.

## Results

### Growth Kinetics and Virulence of *V. anguillarum* RV22 at Cold (15°C) and Warm (25°C) Temperatures

**FIGURE 1:**
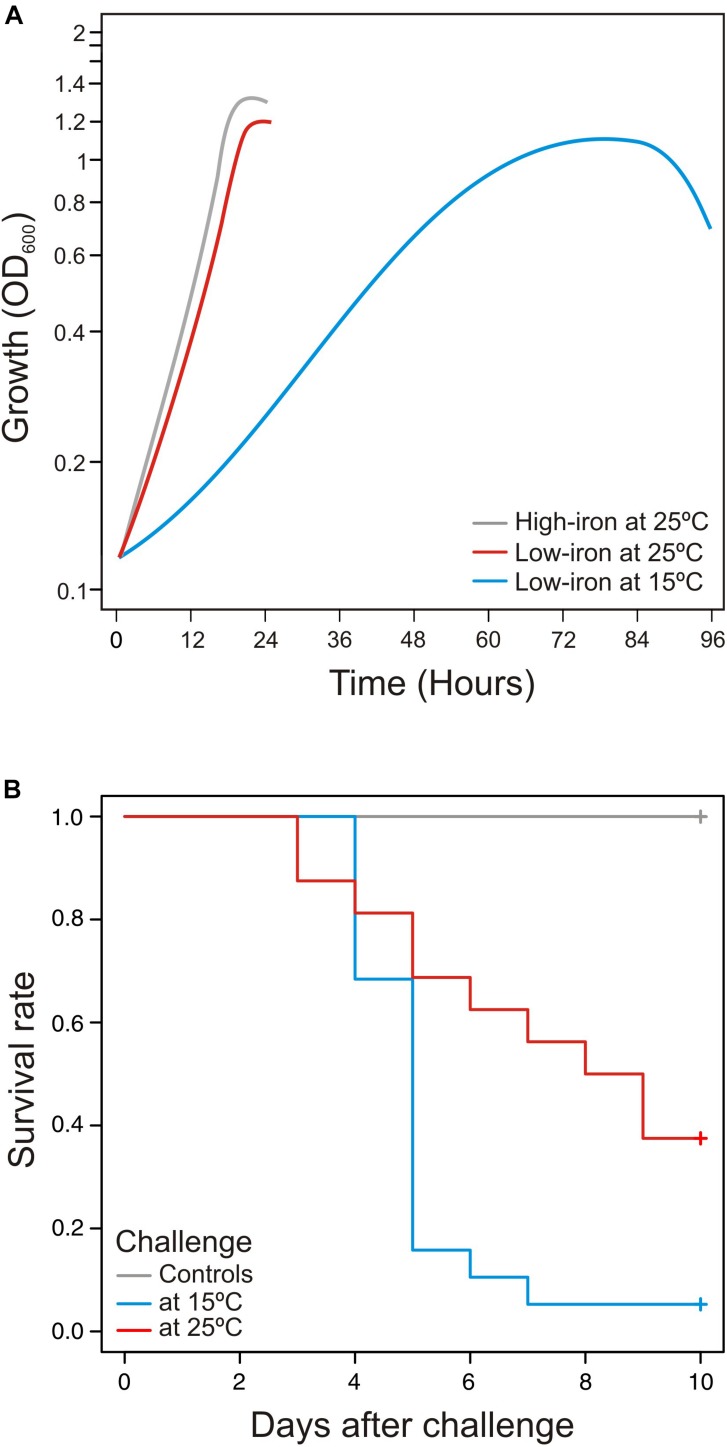
**(A)** Influence of temperature in growth dynamics and **(B)** virulence of *Vibrio anguillarum* at cold-water (15°C) and warm-water (25°C) temperature. Growth **(A)** and virulence **(B)** of *V. anguillarum* RV22 strain assayed at two different temperatures: 15 and 25°C. Growth was assayed in CM9 minimal medium supplemented with Fe_2_SO_4_ 10 μM (high-iron conditions) or 2,2′-dipyridyl 50 μM (low-iron conditions) by measuring OD_600_ for 24 h (25°C) or 96 h (15°C). Senegalese sole fingerlings were experimentally infected with the same dose of *V. anguillarum* RV22 (2–5 × 10^5^ CFU per fish) and kept at 15 or 25°C for 10 days, recording mortalities daily.

Temperature is a determining factor that could have effects on immune response of ectotherms, especially if fish are exposed to temperatures outside their thermal limits (Fletcher and Secombes, 2015). Thereby, in order to assay the virulence properties of *V. anguillarum* at two different water temperatures (15 and 25°C), *S. senegalensis* was chosen as a fish model, since it is a species that is naturally exposed to water temperatures from 12 to 26°C (Arjona et al., 2010). Then, survival curves at each temperature were compared to the growth ability of *V. anguillarum* when it is cultivated in CM9 medium (Figure 1A). Groups of 30 fish were acclimated at water temperature of 15°C (cold) or 25°C (warm) and infected with the same dose of *V. anguillarum* RV22 (2–5 × 10^5^ CFU per fish). No mortality was observed in fish control groups inoculated with 0.1 ml of saline solution (0.85% NaCl). The results show that the fish mortality observed after infection with *V. anguillarum* RV22 significantly increase (*p* = 0.006) at cold-water temperature (Figure 1B). Mortality observed at 15°C was higher than that found at 25°C, reaching 95% 7 days post-infection. On the contrary, when the fish were maintained at a temperature of 25°C, a 63% mortality was reached 9 days after challenge. Thus, the slow growth of *V. anguillarum* when cultivated *in vitro* at 15°C (Figure 1A) greatly contrasts with the substantial increase in its virulence at the same temperature compared with the virulence at 25°C (Figure 1B).

### Analysis of Transcriptome Changes at 25 and 15°C Under Low-Iron Conditions

**FIGURE 2:**
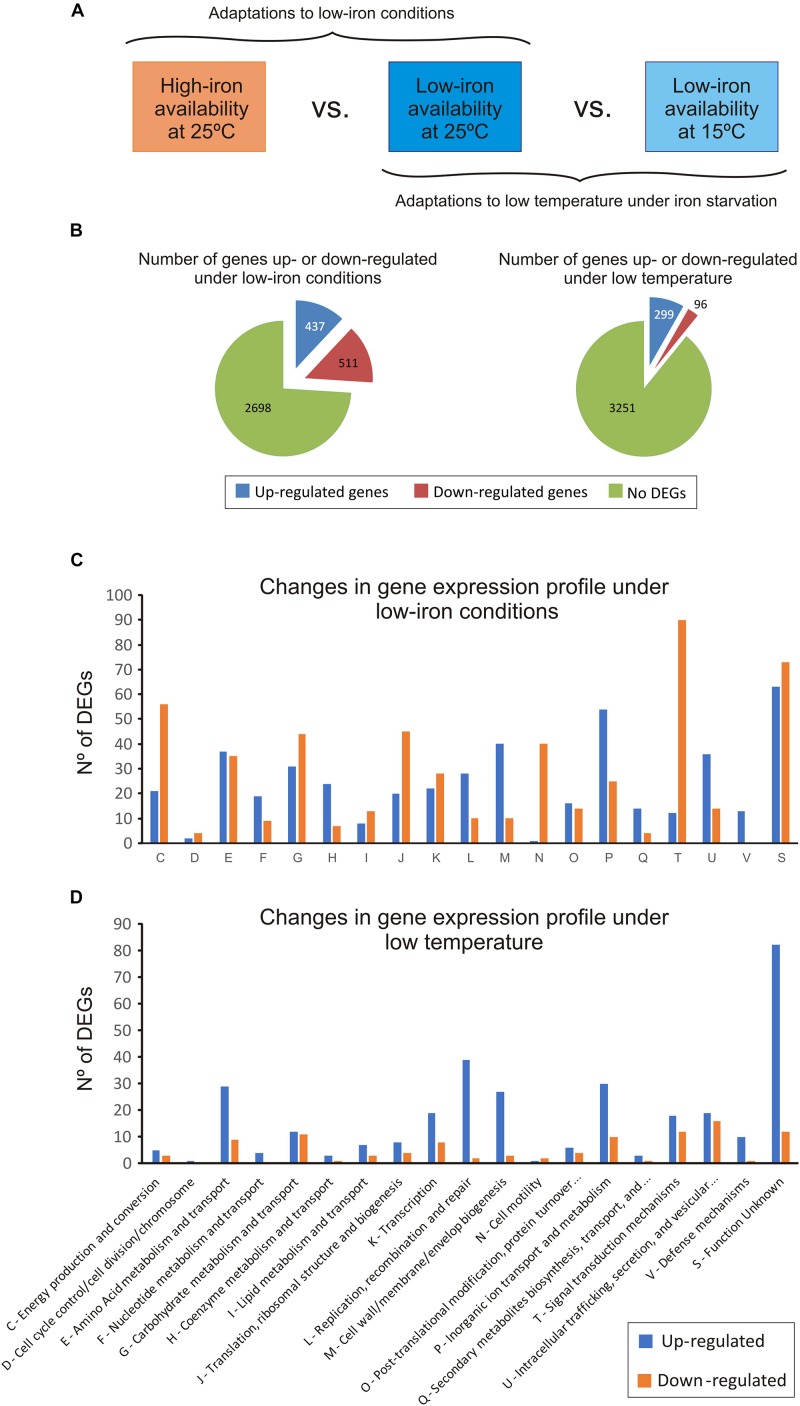
Changes in the transcriptome of *Vibrio anguillarum* RV22 between low- and high-iron conditions at 25°C (to test the effect of iron) and between 25 and 15°C under iron starvation (to test the effect of temperature). **(A)** General overview of the experimental design. **(B)** Summary of the number of differentially expressed genes (DEGs), up-regulated and down-regulated, under iron limitation and at low temperature. **(C)** Number of DEGs under iron limitation classified under the KEGG categories. **(D)** Number of DEGs, up-regulated and down-regulated, at 15°C classified under the KEGG categories.

To analyze the expression pattern of *V. anguillarum* during iron starvation at cold- and warm-water temperature, an RNA-Seq assay was performed in cells of *V. anguillarum* RV22 grown in CM9 minimal medium, supplemented with 50 μM of the iron chelator 2,2′-dipyridyl (low-iron conditions), at 15 and 25°C (Figure 2A). The addition of 2,2′-dipyridyl reduces the availability of iron, mimicking the iron-deprived conditions that bacterial pathogens find during host colonization (Skaar, 2010). In addition, we used as a control the expression pattern of *V. anguillarum* RV22 grown at 25°C in CM9 supplemented with iron (high-iron conditions).

The comparative transcriptome analysis under low- and high-iron at 25°C identified 948 differentially expressed genes (DEGs) (Figure 2B): 511 genes were down-regulated and 437 were up-regulated under low-iron conditions. The complete data set is shown in Supplementary Table S2. For a better interpretation of the low-iron induced adaptations, the predicted products of DEGs were grouped in 13 functional KEGG categories (Figure 2C). The results showed profound changes in cellular metabolism. Most of the down-regulated DEGs encode functions related to signal transduction (category T), energy metabolism (C), cell motility (N), and translation and ribosome structure (J). By contrast, functions related to DNA replication, recombination and repair (L), cell wall/membrane biogenesis (M), and inorganic ion transport (P) were up-regulated under low iron. In addition, the number of DEGs down- and up-regulated related to amino acids (E) and carbohydrate metabolism (G) functions, as well as some transcriptional factors (K) were equilibrate, which would suggest that the adaptation to grow under iron deprivation implies changes in amino acid and carbohydrate requirements (Figure 2C). Specific aspects of low-iron adaptation are detailed below.

Most DEGs at low temperature were up-regulated (299 DEGs) (Figures 2B,D). They were mostly related with amino acids metabolism and transport (E), DNA replication recombination and repair (L), cell wall/membrane biogenesis (M), and inorganic ion transport (P) (Figure 2D). Despite the significantly slow growth rate at 15°C, the expression pattern of the genes related to the energetic metabolism was quite similar. There was a down-regulation of some metabolic genes including the regulator HexR, a protein that mediates the control of the central carbon metabolism by repression (Leyn et al., 2011). In addition, a pronounced down-regulation of genes involved in the synthesis of large and small subunits of ribosomes and tRNA biogenesis (translation, J) was observed. Thus, these limiting conditions required a low level of ribosomal proteins for the bacterial cell and demand a low rate of polypeptide synthesis (Dennis and Bremer, 2008). Some components of the carbohydrate and amino acid transport systems (ABC transporters and phosphotransferase systems) were also down-regulated at 15°C (Supplementary Table S2). The results suggest that the decrease of nutrient import to the cell under the conditions tested negatively affects the ability of *V. anguillarum* RV22 to grow at low temperatures, resulting in a slower growth kinetics.

### Iron Starvation Redirects Central Metabolism and Induces the Expression of Virulence Factors

The analysis of metabolic genes up- and down-regulated under iron starvation showed global metabolic adaptations and the induction of most virulence factors (Supplementary Table S3). In particular, genes encoding functions of TCA cycle and cytochromes were down-regulated extensively. The ATP synthase was also down-regulated. By contrast, alternative members of the respiratory chain were up-regulated. Simultaneously, seven DEGs coding enzymes of the glycolytic pathway and most genes coding pentose phosphate pathway functions were up-regulated. Under iron starvation genes encoding functions for valine, leucine and isoleucine degradation were down-regulated. The genes *hutGHIU* involved in histidine metabolism were also down-regulated. By contrast, biosynthesis of the aromatic amino acids phenylalanine, tyrosine, and tryptophan were up-regulated since the operon *trpBCGE* was 4- to 6-fold induced. However, conversion of phenylalanine to tyrosine was repressed since transcription of *phhA* decreased. Additionally, the synthesis pathway of arginine (arginine/ornithine), which is one of the amino acids required to synthesize the siderophore vanchrobactin (Soengas et al., 2006), was induced 2-fold.

The virulence-related factors identified to date in *V. anguillarum* include LPS, motility and chemotaxis, multiple iron-uptake systems, and the secretion of extracellular products with hemolytic and proteolytic activities (Li and Ma, 2017; Toranzo et al., 2017). Most genes involved in polysaccharide production (VAR_RS0112290–VAR_RS0112465), transport and assemble (*wza–wzc*) (VAR_RS0107285–VAR_RS0107295), and *wbfB–wbfD* (VAR_RS0107305–VAR_RS0107315) (Croxatto et al., 2007; Naka et al., 2011) were significantly up-regulated during iron starvation (Supplementary Table S4). In this regard, we tested biofilm formation under the same conditions used for RNA-Seq, and no significant differences were detected between cells grown under iron starvation or under iron excess at 25°C (Supplementary Figure S1). Hemolytic activity is a major virulence factor in *V. anguillarum* and contributes to the hemorrhagic septicemia characteristic of vibriosis (Hirono et al., 1996). Some cytotoxic proteins have been characterized to date including EmpA, Vah1 cluster (*vah1* and *plp* genes), Vah2–5 hemolysins, and the multifunctional autoprocessing Rtx toxin (MARTX, *rtxACHBDE*) (Li et al., 2008). The genome of *V. anguillarum* RV22 contains *vah1–3* and MARTX toxins (does not contain either *empA*, *vah4*, or *vah5* gene). It is noteworthy that Vah1 and Vah2 hemolysins showed high expression levels under high-iron conditions, which greatly contrast with the low expression level of MARTX and Vah3 under the same condition. While Vah2 was almost constitutively expressed in all assayed conditions, Vah1, Vah3, and MARTX were induced when *V. anguillarum* was grown under low iron (Table 1 and Supplementary Table S5). Some DEGs up-regulated under low iron were identified as outer membrane transporters. They include genes encoding non-specific outer membrane proteins (OMP proteins) and two complete Type VI Secretion Systems (T6SS).

**TABLE 1:** Expression of most relevant virulence-related factors at 25 and 15°C and under iron deficiency.

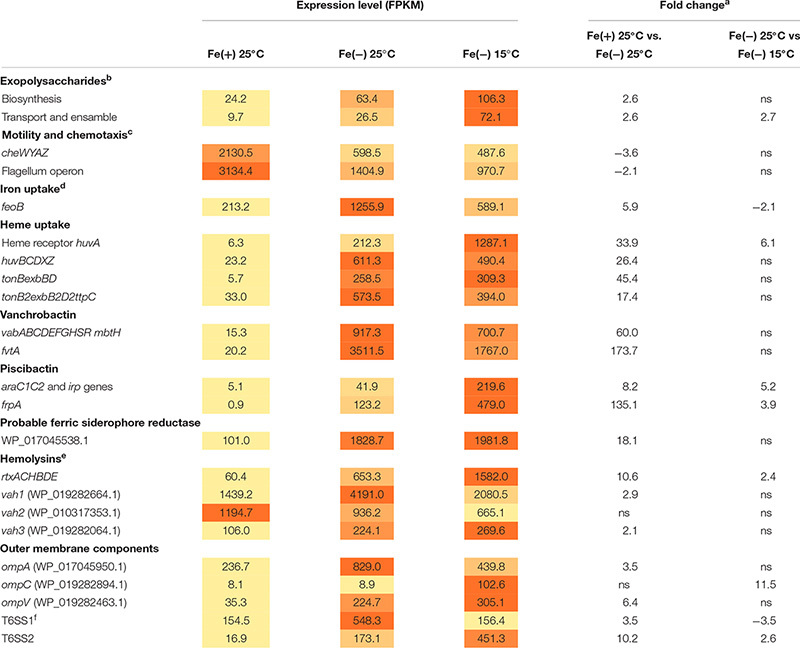

*Darker color shadows denote higher expression. ^*a*^Only fold change values with *p* < 0.05 are shown. ns denotes that non-significant differences were found. ^*b*^Detailed expression for each gene is shown in Supplementary Table S4. ^*c*^Detailed expression for each gene is shown in Supplementary Table S6. ^*d*^Detailed expression for each gene is shown in Supplementary Table S8. ^*e*^Detailed expression for each gene is shown in Supplementary Table S5. ^*f*^Detailed expression for each gene is shown in Supplementary Table S7.*

Not surprisingly, iron acquisition systems were strongly up-regulated under iron starvation. The *feoB* that mediates ferrous iron acquisition (Li and Ma, 2017) was 5.9-fold induced. *huvABCDZX* genes encoding heme uptake and utilization system (Mouriño et al., 2004) increased the transcription level more than 30-fold and, more notably, the genes encoding siderophore systems, vanchrobactin and piscibactin, were among the top up-regulated genes since they were induced more than 60-fold. Nonetheless, at 25°C, the vanchrobactin system is expressed more than 20-fold compared to piscibactin. Both ferri-siderophore complexes and heme group are internalized via specific outer membrane receptors in a process that is driven by the inner membrane potential and mediated by the energy-transducing systems TonB1 and TonB2, whose genes were up-regulated 46- and 18-fold under iron starvation. Interestingly, locus VAR_RS0103565 was induced 18.1-fold under iron starvation and it could code a putative ferric-siderophore transport protein uncharacterized to date.

Overall, virulence-related factors were up-regulated under low iron compared to high iron either at 25 or 15°C, although two exceptions were found. Chemotaxis-related (*cheWYAZ*) and motility-related (*fli* and *flg*) genes decreased their expression 2.3- and 5.8-fold when *V. anguillarum* was grown under low iron (Supplementary Table S6). In agreement with this observation, the evaluation of motility in soft agar showed that *V. anguillarum* RV22 is less motile under iron starvation (Supplementary Figure S2).

### *V. anguillarum* Expresses a Specific Cocktail of Virulence Factors at Each Temperature, Either 25 or 15°C

The main focus of the present work was to characterize the changes in expression of virulence factors after temperature drop that would allow the understanding of the enhanced virulence shown by *V. anguillarum* at 15°C compared to 25°C water temperature. RNA-Seq results showed that, although the expression of LPS biosynthetic genes did not show statistically significant differences, genes encoding functions related to exopolysaccharide synthesis showed maximal expression at low temperature (Supplementary Table S4). In fact, biofilm formation showed a statistically significant increase at 15°C (Supplementary Figure S1). As shown above, MARTX is almost silenced under high-iron conditions, increasing 10-fold the operon transcription when *V. anguillarum* senses low-iron levels. Besides, the expression of MARTX was 2.4-fold up-regulated at 15°C. Thus, MARTX would be preferentially expressed at low temperatures and under iron starvation (Table 1 and Supplementary Table S5). The effect of temperature on hemolytic phenotype could be detected on Columbia Agar plates, where *V. anguillarum* RV22 cells caused a higher translucency of the hemolytic halo when it was grown at 15°C (Supplementary Figure S3), indicating production of different hemolysins at either 25 or 15°C.

While major porines OmpA and OmpV were 3.5- and 6.4-fold induced under iron starvation at 25°C, transcription of *ompC* increased only at low temperature (11.5-fold). *Vibrio anguillarum* genome harbors two T6SS (Guanhua et al., 2018). T6SS1 and T6SS2 were induced 3- and 10-fold, respectively, under iron starvation. However, T6SS1 was ca. 3-fold more expressed at 25°C than T6SS2 (Table 1 and Supplementary Table S7). It is noteworthy that each T6SS responds to low temperature in opposite ways. While T6SS1 was 3.5-fold down-regulated (decrease from 550 to 156), T6SS2 was 2.6-fold induced (Table 1). Thus, both systems are up-regulated during iron starvation, but T6SS1 is preferentially expressed at 25°C and T6SS2 at 15°C. Overall, the results showed that T6SS1 has a high basal expression level since it was significantly expressed in all the assayed conditions. By contrast, T6SS2 required low iron and cold temperature to be induced.

**FIGURE 3:**
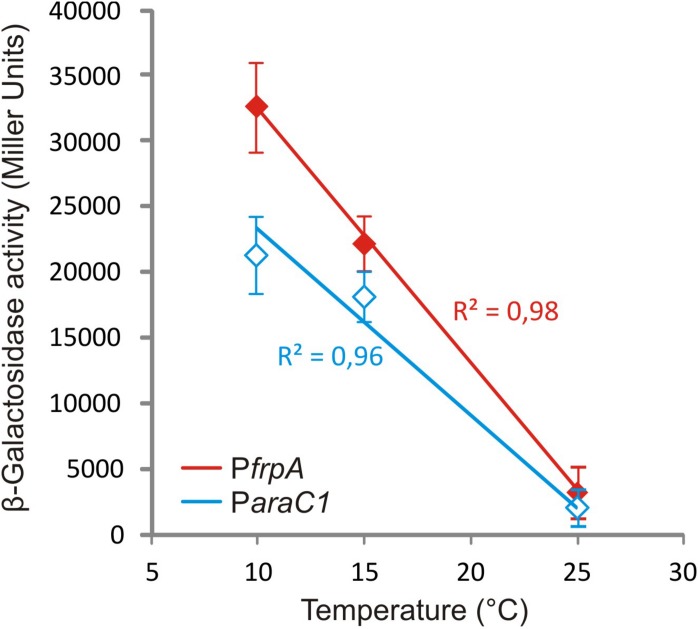
Plot of β-galactosidase activity of transcriptional fusions between two promoters from siderophore piscibactin gene cluster (P*frpA* and P*araC1*) of *Vibrio anguillarum* RV22 and *lacZ* at increasing temperatures. All measures were carried out in triplicate, and standard deviations for each point are indicated.

Piscibactin biosynthetic and uptake genes as well as heme receptor HuvA were greatly up-regulated at low temperature. Regarding the piscibactin system, the AraC-like transcriptional regulator *araC1* (VAR_RS0103450) and the putative piscibactin outer membrane receptor *frpA* (VAR_RS0103460) were two of the most up-regulated genes at 15°C since their transcription showed a 6.8- and 3.9-fold increase, respectively (Table 1 and Supplementary Table S8). To confirm whether temperature acts as an environmental regulatory signal that modulates piscibactin gene transcription, the transcriptional activities of *frpA* (P*frpA*) and *araC1* (P*araC1*) (Balado et al., 2018) promoters were measured at three temperatures (10, 15, and 25°C). The results indicate that the β-galactosidase activity of both piscibactin promoters dramatically decreased as temperature was increased (Figure 3), denoting a strong negative correlation between temperature and the transcriptional activity of piscibactin promoters. These results confirm the transcriptomic data and demonstrate that piscibactin operon is repressed at 25°C and that it is highly expressed at low temperatures.

## Discussion

The water temperature is a risk factor that enhances the occurrence of disease outbreaks caused by *Vibrio* species (Le Roux et al., 2015). *V. anguillarum* is able to cause disease outbreaks in a wide temperature range, since it can infect many different farmed fish species at both cold- and warm-water temperatures (Bellos et al., 2015; Ma et al., 2017; Rønneseth et al., 2017; Toranzo et al., 2017). On the other hand, pathogenic bacteria face a drastic shift in iron availability upon host encounter (Skaar, 2010). Low-iron adaptations were described in most bacterial pathogens, and it is well established that low-iron conditions up-regulate the expression of most virulence factors (Andrews et al., 2003; Mey et al., 2005; Friedman et al., 2006). Our results show that both environmental signals, iron and temperature, dually regulate the expression of virulence factors and other key components of the cell. We have shown that although a low temperature reduces the growth of *V. anguillarum*, the virulence achieved for fish kept in cold water (15°C) is significantly higher than that observed in warmer water (25°C). Although low temperature can have negative effects on the fish immune system, especially on the adaptive immunity (Fletcher and Secombes, 2015), the observation that, at 15°C, there is an increase in the expression of some relevant virulence factors such as the heme receptor HuvA, siderophore piscibactin, MARTX toxins, and T6SS2, could explain in part the higher virulence at this temperature. However, other important factors like hemolysin Vah1, T6SS1, ferrous iron transport, and vanchrobactin siderophore system showed higher expression at the optimal growth temperature (25°C). Thus, all these suggest that *V. anguillarum* produces a specific cocktail of virulence factors at each temperature and that its virulence at a particular temperature would be more related to the modulation of expression of virulence factors than to the higher or lower capacity to grow.

Numerous virulence factors have been described in *V. anguillarum* (Toranzo et al., 2017; Hickey and Lee, 2018), and although most studies were performed in strains from serotype O1, some discrepancies exist among serotypes (Tang et al., 2016). *V. anguillarum* LPS have endotoxic activity and allow the pathogen to adhere and to persist within the host by evading the immune system response (Boesen et al., 1999; Lindell et al., 2012). We found a significant up-regulation of genes involved in LPS biosynthesis, assembly, and export under iron starvation conditions, but additionally, their expression increased at low temperature (Supplementary Table S4). Chemotaxis and motility have been recognized as virulence-related factors (Larsen, 2004; Guanhua et al., 2018). Although *V. anguillarum* RV22 is motile under the three conditions tested (Supplementary Figure S2), our results showed that chemotaxis and flagellum-related genes were significantly down-regulated under both low iron availability and low temperature. Low-iron conditions also reduced motility of *Vibrio vulnificus* (Pajuelo et al., 2016). Nevertheless, although genes encoding structural components of flagellum were down-regulated, their basal expression was relatively high, and some proteins of the flagellum motor were even up-regulated under those conditions. Congruently, it was reported that *V. anguillarum* is chemotactic and motile at the temperature range of 5–25°C, with the maximal motility achieved at 25°C (Larsen, 2004). Active motility is needed for entry into the fish host (Ormonde et al., 2000), but once the bacterium has invaded the fish, motility is no longer needed for the progression of vibriosis (Milton et al., 1996; O’Toole et al., 1996).

Hemolysin Vah1 and MARTX toxin are mainly responsible for the hemolytic and cytotoxic activities of *V. anguillarum* in fish (Hirono et al., 1996; Li et al., 2008). Vah1 and RtxA have cytotoxic effects on Atlantic salmon kidney cells and only a *vah1 rtxA* double mutant was no longer cytotoxic. However, only *rtxA* mutants had reduced virulence in Atlantic salmon (Li et al., 2008). Our results revealed that MARTX is preferentially produced at 15°C. Consequently, the implication of MARTX in the virulence assays previously reported could be overestimated, since the implication of MARTX in virulence would vary according to the environment temperature.

Three non-specific outer membrane proteins (OMP proteins) were differentially expressed at 15 and 25°C. While OmpA and OmpV were induced under iron starvation, expression of OmpC significantly increased under iron deficiency but only at 15°C (Table 1). OmpA plays a crucial role in the attachment of bacterial cells to the host cells during the invasion process and evasion of host defenses (Confer and Ayalew, 2013). OmpV is one of the major outer membrane proteins in *V. cholerae* and it is highly immunogenic (Stevenson et al., 1985). OmpC is involved in membrane integrity maintenance and resistance to antibiotics (Choi and Lee, 2019) and it has been reported that its synthesis is favored in high osmolarity conditions and warm temperatures (25°C) (Davey et al., 1998).

*Vibrio anguillarum* has two T6SS that respond to iron deprivation by increasing their expression. In many bacteria, two or more T6SS gene clusters are found in a single bacterial genome (Bingle et al., 2008), often possessing different functions and regulation mechanisms (Cascales, 2008; Bernard et al., 2010; Schwarz et al., 2010). Interestingly, our results show that while T6SS1 is preferentially expressed at 25°C, T6SS2 shows the highest expression at 15°C. Although no significant loss of virulence was observed in *V. anguillarum* when T6SS2 was inactivated (Weber et al., 2009; Tang et al., 2016), recent works showed that inactivation of either T6SS1 or T6SS2 decreased the fitness of the bacterium within the fish (Guanhua et al., 2018). It was also reported that T6SS2 would regulate the expression of extracellular proteases via the stress-response regulator RpoS and the *quorum sensing* regulator VanT (Weber et al., 2009). Notably, the expression pattern of T6SS2 genes showed large discrepancies between O1 and non-O1 strains harboring 100% identical gene clusters. In some strains, their expression was not detected, while in other strains, its expression was more active under warm marine-like conditions (Tang et al., 2016). These findings suggest that unidentified expression factor(s) encoded outside the T6SS cluster might modulate its expression. The functional impact of T6SS in *V. anguillarum* fitness needs to be elucidated in further studies.

The genes encoding vanchrobactin and piscibactin siderophore systems are among the most up-regulated genes under iron starvation, increasing more than 60-fold their transcription. Nonetheless, the vanchrobactin system is significantly more expressed than piscibactin at 25°C. The results proved that the expression of P*araC1* and P*frpA* promoters, which control the transcription of piscibactin genes (Balado et al., 2018), is proportional to the temperature decrease. Thus, while piscibactin genes are almost silenced at 25°C, they are significantly transcribed at 15°C. This result reinforces previous work showing that piscibactin siderophore has a dual requirement of low iron and low temperature to be synthesized (Balado et al., 2018). Since piscibactin is more relevant for virulence than vanchrobactin (Balado et al., 2018), the piscibactin transcription increment at 15°C would partially explain the higher degree of virulence of *V. anguillarum* at this temperature.

## Conclusion

Knowledge on virulence factors regulation is essential for the development of new immunoprophylactic and chemotherapeutic treatments against the bacterial infections (Cole et al., 2009). Overall, the results obtained in the present work showed that disease severity caused by *V. anguillarum* is multifactorial and context dependent. *V. anguillarum* modulates the expression of most virulence factors in a temperature-dependent manner and in response to low iron levels. Thus, each virulence factor is favored at a specific temperature range, which implies that its relevance could greatly vary among the different fish species that this bacterium can infect, and even among different geographic locations. Current fish vaccines against vibriosis are usually produced by growing the bacterium at 25°C and in rich media with an excess of iron. Under these conditions, according to our results, some relevant cell components may not be adequately expressed, resulting in a vaccine that lacks some important antigens that are produced by *V. anguillarum* during an infection, which could result in vaccines with a lower degree of protection. Altogether, our results give clues about the physiological adaptations that allow *V. anguillarum* to cause infections in different fishes and could be useful to implement novel strategies to combat the incidence of vibriosis outbreaks by optimization of vaccine preparations.

## Data Availability Statement

The datasets generated for this study can be found in the NCBI Sequence Read Archive (SRA). Accession Number: SRP213600.

## Ethics Statement

The animal study was reviewed and approved by Bioethics Committee of the University of Santiago de Compostela.

## Author Contributions

MAL and MB performed the lab experiments, analyzed the data, and wrote the first draft of the manuscript. MB and MLL corrected the draft and built the final version of the manuscript. All authors conceived and designed the study, contributed to manuscript revision, and read and approved the submitted version.

## Conflict of Interest

The authors declare that the research was conducted in the absence of any commercial or financial relationships that could be construed as a potential conflict of interest.
