# Supplementary material for: The Expression of Virulence Factors in Vibrio anguillarum Is Dually Regulated by Iron Levels and Temperature
Source: Front Microbiol. 2019 Oct 15;10:2335. doi: 10.3389/fmicb.2019.02335 (PMC6803810; doi:10.3389/fmicb.2019.02335)
Supplement: Supplementary file 1 [file Table_1.pdf]

Supplementary Material

The expression of virulence factors in *Vibrio anguillarum* is dually regulated by iron levels and temperature

Marta A. Lages, Miguel Balado, Manuel L. Lemos*

Department of Microbiology and Parasitology, Institute of Aquaculture, Universidade de Santiago de Compostela, Santiago de Compostela 15782, Spain.

Supplementary Table S1. Reads mapped statistics.

Supplementary Table S2. Excel Data Sheet under separate file. Complete data of genes differentially expressed at 25 ºC under iron excess and under iron deficiency (Sheet 1). Complete data of genes differentially expressed at 25 ºC and at 15ºC under iron deficiency (Sheet 2).

Supplementary Table S3. Relevant metabolism-related genes differentially expressed at 25 ºC and 15 ºC and under iron deficiency.

Supplementary Table S4. Expression of LPS related genes at 25 ºC and 15 ºC and under iron deficiency.

Supplementary Table S5. Expression of haemolysins at 25 ºC and 15 ºC and under iron deficiency.

Supplementary Table S6. Expression of chemotaxis and motility related genes at 25 ºC and 15 ºC and under iron deficiency.

Supplementary Table S7. Expression of type VI secretion systems (T6SS) and outer membrane permeases at 25 ºC and 15 ºC and under iron deficiency.

Supplementary Table S8. Expression of heme uptake and siderophore systems at 25 ºC and 15 ºC and under iron deficiency.

Supplementary Figure S1. Biofilm formation of *V. anguillarum* RV22 at 25 ºC under high- and low-iron conditions and at 15 ºC under low iron.

Supplementary Figure S2. Motility of *V. anguillarum* RV22 at 25 ºC under high- and low-iron conditions and at 15 ºC under low iron.

Supplementary Figure S3. Haemolytic activity of *V. anguillarum* RV22 at 25 ºC and 15 ºC.

Table S1. Reads mapped statistics.

Sample	SRA sample id.	Input reads	Reads mapped (%)	Multiple alignments reads (%)
Fe(+) at 25 ºC sample A	SRR9651603	24963269	17634777 (70.6%)	238798 (1.4%)
Fe(+) at 25 ºC sample B	SRR9651604	27276794	23180570 (85.0%)	274730 (1.2%)
Fe(+) at 25 ºC sample C	SRR9651600	25715318	20411474 (79,4%)	23517 (0.1%)
Fe(-) at 25 ºC sample A	SRR9651602	25973584	19498931 (75.1%)	89741 (0.5%)
Fe(-) at 25 ºC sample B	SRR9651598	28816113	21334235 (74.0%)	49320 (0.2%)
Fe(-) at 25 ºC sample C	SRR9651599	25867855	21613361 (83.6%)	34290 (0.2%)
Fe(-) at 15 ºC sample A	SRR9651597	23882118	19137111 (80.1%)	174156 (0.9%)
Fe(-) at 15 ºC sample B	SRR9651601	28469912	19520666 (68.6%)	194412 (1.0%)
Fe(-) at 15 ºC sample C	SRR9651605	27063524	23632512 (87,3%)	511877 (2.2%)

RNA-Seq reads were deposited at SRA database (Sequence Read Archive) under study id. SRP213600.

Table S3. Relevant metabolism-related genes differentially expressed at 25 ºC and 15 ºC and under iron deficiency. Darker colour shadows denote higher expression.

Gene	Description	Expression level (FPKM)			Fold change[a]	
		Fe(+) 25ºC	Fe(-) 25ºC	Fe(-) 15ºC	Fe(+) 25ºC vs Fe(-) 25ºC	Fe(-) 25ºC vs Fe(-) 15ºC
Central metabolic regulator						
iscR (WP_026027698.1)	DNA-binding transcription factor	394.841	1701.96	1981.35	4.3	ns
erpA (WP_026027518.1)	Iron-sulfur cluster insertion protein	84.1258	198.739	342.876	2.4	ns
hexR (WP_019281735.1)	Represor of central carbon metabolism	9.79078	10.442	40.3567	ns	3.9
hscA (WP_019282106.1)						
Glycolysis						
pgm (WP_019281944.1)	Phosphoglucomutase	43.9	189.5	222.3	4.3	ns
pfkA (WP_029388378.1)	6-phosphofructokinase	380.9	1341.1	1335.1	3.5	ns
fbaA (WP_013857804.1)	Fructose-bisphosphate aldolase	355.5	744.4	621.4	2.1	ns
gapdH (WP_010317219.1)	Glyceraldehyde-3-phosphate dehydrogenase	682.7	1716.5	1942.2	2.5	ns
pgk (WP_026027969.1)	Phosphoglycerate kinase	336.5	1068.9	1006.5	3.2	ns
gpmI (WP_019282807.1)	2,3-biphosphoglycerate-independent phosphoglyceromutase	215.7	1124.0	811.6	5.2	ns
pykF (WP_029388154.1)	Pyruvate kinase	202.5	448.2	338.2	2.2	ns
TCA cycle						
gltA (WP_019281581.1)	Citrate (Si)-synthase	161.6	51.5	125.0	-3.1	2.4
mdh (WP_017043806.1)	Malate dehydrogenase	1498.4	631.1	803.8	-2.4	ns
fumA (WP_029388383.1)	Fumarate hydratase	565.0	211.3	249.5	-2.7	ns
sdhA (WP_013856347.1)	Succinate dehydrogenase flavoprotein subunit	2750.5	616.5	505.4	-4.5	ns
sdhB (WP_013856348.1)	Succinate dehydrogenase iron-sulfur subunit	729.2	139.4	121.2	-5.2	ns
sdhC (WP_019281580.1)	Succinate dehydrogenase cytochrome b556	831.0	87.9	50.4	-9.5	ns
sdhD (WP_010318675.1)	Succinate dehydrogenase, anchor protein	2088.6	241.8	151.6	-8.6	ns
frdABCD	Fumarate reductase	531.0	58.5	50.0	-8.3	ns
frdA (WP_013855868.1)	Fumarate reductase flavoprotein subunit	712.5	90.2	104.1	-7.9	ns

frdB (WP_017046770.1)	Fumarate reductase iron-sulfur subunit	453.2	86.6	51.5	-5.2	ns
frdC (WP_017043204.1)	Fumarate reductase subunit C	360.2	44.1	34.4	-8.2	ns
frdD (WP_010317640.1)	Fumarate reductase subunit D	156.0	13.2	10.0	-11.8	ns
sucB (WP_019281578.1)	Dihydrolipoamide succinyltransferase	973.3	392.7	283.3	-2.5	ns
Pentose phosphate pathway						
zwf (WP_029388423.1)	Glucose-6-phosphate dehydrogenase	30.9	175.2	282.4	5.7	ns
gnd (WP_019283138.1)	6-phosphogluconate dehydrogenase	33.3	303.8	468.3	9.1	ns
fbaA (WP_013857804.1)	Fructose-bisphosphate aldolase	355.5	744.4	621.4	2.1	ns
glpX (WP_019282901.1)	Fructose 1,6-bisphosphatase	69.8	153.3	125.9	2.2	ns
pfkA (WP_029388378.1)	6-phosphofructokinase	380.9	1341.1	1335.1	3.5	ns
Histidine metabolism						ns
hutH (WP_017048047.1)	Histidine ammonia-lyase	65.4	8.8	11.4	-7.4	ns
hutU (WP_019283311.1)	Urocanate hydratase	50.4	6.5	15.7	-7.8	2.4
hutI (WP_029388451.1)	Imidazolonepropionase	19.1	1.8	6.1	-10.5	ns
hutG (WP_029388450.1)	Formimidoylglutamase	24.0	1.6	3.4	-14.6	ns
Fatty acid metabolism						
fadA (WP_019281690.1)	Fatty acid oxidation complex					
fadB (WP_019281689.1)	Fatty acid oxidation complex subunit alpha	247.2	33.3	38.3	-7.4	ns
fadD (WP_017629527.1)	Fatty acid oxidation complex					
fadE (WP_019282302.1)	Fatty acid oxidation complex					
fadJ (WP_019283103.1)	Fatty acid oxidation protein subunit alpha	318.8	62.4	93.3	-5.1	ns
fadI (WP_019283104.1)	Fatty acid oxidation complex					
Valine, leucine and isoleucine degradation						
lpd (WP_026027892.1)	Dihydrolipoamide dehydrogenase	4.0	0.9	1.8	-4.4	ns
fadI (WP_019283104.1)	3-ketoacyl-CoA thiolase	275.1	62.5	83.3	-4.4	ns
ascA (WP_010319642.1)	Acetoacetyl-CoA synthetase	86.3	28.1	28.6	-3.1	ns
Arginine biosynthesis						
aspC (WP_019282378.1)	Aspartate aminotransferase	360.1	685.7	879.7	1.9	ns
argA (WP_026027647.1)	Amino-acid N-acetyltransferase	122.5	281.2	448.0	2.3	ns
argB (WP_010317663.1)	Acetylglutamate kinase	626.9	1206.1	2213.7	1.9	ns
argC (WP_019282443.1)	N-acetyl-gamma-glutamyl-phosphate reductase	420.3	1096.0	1610.6	2.6	ns
argG (WP_017043213.1)	Argininosuccinate synthase	1719.3	4046.0	5666.2	2.4	ns
Phenylalanine, tyrosine and tryptophan biosynthesis						

trpE (WP_019281982.1)	Anthranilate synthase subunit I	77.6	380.6	149.7	4.9	-2.5
trpG (WP_019281983.1)	Amidotransferase of anthranilate synthase	136.8	908.7	174.4	6.6	-5.2
trpC (WP_019281984.1)	Phosphoribosylanthranilate isomerase	62.4	365.5	213.5	5.9	ns
trpB (WP_013857145.1)	Tryptophan synthase, beta subunit	683.4	4147.6	1021.0	6.1	-4.1
trpR (WP_013857539.1)	*trp* operon repressor	230.221	450.574	435.671	2.0	ns
phhA (WP_019282676.1)	Phenylalanine 4-monooxygenase	80.3	39.0	29.5	-2.1	ns
tyrA (WP_017043283.1)	Chorismate mutase	272.8	561.8	322.7	2.1	ns
nadB (WP_017046483.1)	L-aspartate oxidase	84.3	283.9	314.6	3.4	ns
Respiratory chain						
WP_019281580.1	Cytochrome b556	831.0	87.9	50.4	-9.5	ns
WP_019281630.1	Cytochrome C	426.2	15.7	17.5	-27.2	ns
WP_013855655.1	Cytochrome C	143.6	67.7	79.4	-2.1	ns
WP_019282134.1	Cytochrome C	1575.6	117.0	89.4	-13.5	ns
WP_026028567.1	Cytochrome C nitrate reductase	60.4	11.1	5.3	-5.4	ns
WP_026027333.1, WP_010320252.1, WP_013856895.1	Cytochrome C oxidase, cbb3-type, subunit I, II and III	3461.0	194.6	174.0	-5.7	ns
WP_026027333.1	Cytochrome C oxidase, cbb3-type, subunit I	194.7	34.3	42.0	-5.7	ns
WP_010320252.1	Cytochrome C oxidase, cbb3-type, subunit II	1490.3	269.5	238.8	-5.5	ns
WP_013856895.1	Cytochrome C oxidase, cbb3-type, subunit III	1776.0	280.1	241.2	-6.3	ns
WP_026027913.1	Cytochrome C biogenesis protein CcsB	292.3	73.9	143.9	-4.0	ns
WP_017046505.1	Cytochrome B	2181.5	363.3	272.1	-6.0	ns
WP_002540812.1	ATP synthase F0, C subunit	589.7	216.3	293.3	-2.7	ns
WP_013855589.1	ATP synthase F0, A subunit	451.7	138.1	198.0	-3.3	ns
WP_013855588.1	F0F1 ATP synthase subunit I	1275.7	532.1	711.7	-2.4	ns
WP_013856176.1	NADH-quinone reductase, F subunit	1261.3	418.3	340.7	-3.0	ns
WP_013856175.1	Ubiquinone oxidoreductase, E subunit	332.8	131.5	100.3	-2.5	ns
WP_005457265.1	Cytochrome BD oxidase subunit I	294.3	600.5	373.1	2.0	ns
rsxB (WP_013857280.1)	Electron transport complex protein RnfB	6.9	27.2	19.5	3.9	ns
rsxE (WP_029388436.1)	Electron transporter RsxE	83.7	201.6	149.3	2.4	ns
rsxC (WP_000949708.1)	Electron transporter RnfC	12.0	48.5	36.3	4.0	ns
rsxD (WP_006963070.1)	Electron transporter RnfG	6.3	21.9	11.1	3.5	ns

[a] Only fold change values with $p < 0.05$ are shown. ns denotes that not significant differences were found.

Table S4. Expression of LPS related genes at 25 ºC and 15 ºC and under iron deficiency. Darker colour shadows denote higher expression.

	Expression level (FPKM)			Fold change[a]	
	Fe(+) 25ºC	Fe(-) 25ºC	Fe(-) 15ºC	Fe(+) 25ºC vs Fe(-) 25ºC	Fe(-) 25ºC vs Fe(-) 15ºC
LPS biosynthesis					
WP_019282749.1	10.3232	23.2583	67.0299	2.3	2.9
WP_019282750.1	28.6925	64.1388	104.614	2.2	ns
WP_019282751.1	23.5349	38.716	61.25	ns	ns
WP_019282752.1	67.7342	119.016	139.861	ns	ns
WP_010317081.1	8.25886	16.686	38.0145	ns	ns
WP_019282753.1	36.9733	86.5046	175.719	2.3	ns
WP_019282754.1	40.5841	100.161	162.17	ns	ns
WP_010317086.1	33.0327	72.7726	126.36	ns	ns
WP_019282755.1	43.3658	173.618	264.417	4.0	ns
WP_019282756.1	44.0879	91.7012	176.738	ns	ns
WP_019282757.1	25.7907	90.9073	128.23	ns	ns
WP_019282758.1	11.7846	32.6429	49.7433	ns	ns
WP_019282759.1	7.76503	10.8502	14.1291	ns	ns
WP_019282760.1	2.45572	8.98473	13.8316	ns	ns
WP_019282761.1	8.89288	30.298	51.1257	3.4	ns
WP_019282762.1	21.6241	65.5437	95.3489	3.0	ns
WP_019282763.1	11.351	41.3446	72.5237	3.6	ns
WP_010317120.1	13.5146	29.2668	55.3569	ns	ns
WP_019282765.1	34.0807	71.2537	141.751	ns	ns
WP_019282766.1	117.277	194.436	290.968	ns	ns
WP_019282767.1	83.7276	166.189	262.383	ns	ns
WP_043004658.1	97.1849	214.714	398.852	2.2	ns
WP_019282768.1	31.22	64.9891	103.531	2.1	ns
WP_029388338.1	9.62516	21.9041	25.758	2.3	ns
WP_019282770.1	0.29613	3.66849	16.2737	ns	4.4
WP_019282771.1	0.76786	1.29479	6.03082	ns	4.7
WP_029388340.1	5.49534	22.6863	55.7805	4.1	2.5
WP_019282773.1	17.7071	36.6227	59.9883	ns	ns
WP_019282774.1	32.6509	73.2255	51.5516	2.2	ns
LPS transport and ensamble					
wzb (WP_029388235.1)	11.6347	28.21	54.8375	2.4	ns
wza (WP_019282252.1)	8.47715	23.5381	81.5519	2.8	3.5
wzi (WP_019282253.1)	22.2108	68.5437	192.299	3.1	2.8
wbfB (WP_019282254.1)	1.00805	2.61944	10.0848	ns	3.8
wbfC (WP_019282255.1)	0.37941	1.37967	5.07576	ns	ns
wbfD (WP_019282256.1)	5.20315	9.65681	21.6635	ns	2.2

[a] Only fold change values with $p < 0.05$ are shown. ns denotes that not significant differences were found.

Table S5. Expression of haemolysins at 25 ºC and 15 ºC and under iron deficiency. Darker colour shadows denote higher expression.

	Expression level (FPKM)			Fold change[a]	
	Fe(+) 25ºC	Fe(-) 25ºC	Fe(-) 15ºC	Fe(+) 25ºC vs Fe(-) 25ºC	Fe(-) 25ºC vs Fe(-) 15ºC
Haemolysins					
vah2 (WP_010317353.1)	1194.68	936.151	665.078	ns	ns
vah1 (WP_019282664.1)	1439.24	4190.97	2080.54	2.9	ns
vah3 (WP_019282064.1)	105.953	224.066	269.647	2.1	ns
MARTX					
rtxA (WP_019282576.1)	35.3543	415.329	1399.13	11.7	3.4
rtxC (WP_010848330.1)	33.3572	418.414	983.098	12.5	2.3
rtxH (WP_013856638.1)	280.614	2960.8	6558.39	10.6	2.2
rtxB (WP_019282577.1)	4.45415	44.0058	180.25	9.9	4.1
rtxC (WP_010319619.1)	5.10154	56.6148	276.16	11.1	4.9
rtxD (WP_017047016.1)	3.22314	24.6371	95.0801	7.6	3.9

[a] Only fold change values with $p < 0.05$ are shown. ns denotes that not significant differences were found.

Table S6. Expression of chemotaxis and motility related genes at 25 °C and 15 °C and under iron deficiency. Darker colour shadows denote higher expression.

	Expression level (FPKM)			Fold change[a]	
	Fe(+) 25ºC	Fe(-) 25ºC	Fe(-) 15ºC	Fe(+) 25ºC vs Fe(-) 25ºC	Fe(-) 25ºC vs Fe(-) 15ºC
Chemotaxis and Motility					
cheW (WP_038170663.1)	2033.18	713.796	466.965	-2.8	ns
WP_026027244.1	256.73	108.131	78.4109	ns	ns
cheY (WP_017045237.1)	701.776	308.252	282.219	-2.3	ns
cheA (WP_019281574.1)	1079.22	388.021	409.818	-2.8	ns
cheZ (WP_017045236.1)	6726.19	2224.29	2189.51	-3.0	ns
cheY (WP_033197405.1)	3960.36	682.6	626.479	-5.8	ns
fliA (WP_010318887.1)	2318.56	493.279	352.914	-4.7	ns
flhG (WP_010318888.1)	1594.56	398.287	333.352	-4.0	ns
flhF (WP_019281575.1)	1204.46	260.841	208.399	-4.6	ns
flhA (WP_013856364.1)	80.239	45.1634	36.9497	ns	ns
sixA (WP_017042529.1)	3480.22	960.556	378.878	-3.6	-2.5
fliE (WP_026028064.1)	1104.24	440.209	339.411	-2.5	ns
WP_026028065.1	82.0278	72.2388	94.2691	ns	ns
WP_013856306.1	74.9664	38.7162	34.272	ns	ns
WP_017045739.1	98.6652	55.1108	44.2178	ns	ns
fliS (WP_010318718.1)	9202.2	1267.04	1278.27	-7.3	ns
fliD (WP_019281598.1)	542.424	202.725	180.483	-2.7	ns
flaG (WP_013856302.1)	41456.6	15525.9	8205.19	-2.7	ns
flaB (WP_026028066.1)	10285.3	3051.42	2738.72	-3.4	ns
flaB (WP_019281599.1)	4917.86	790.794	899.291	-6.2	ns
flaB (WP_019281600.1)	697.139	104.123	157.355	-6.7	ns
fliN (WP_010318705.1)	197.341	104.657	90.697	ns	ns
fliM (WP_013856317.1)	173.746	71.1982	81.0141	-2.4	ns
fliL (WP_000796796.1)	725.779	238.571	164.089	-3.0	ns
WP_017048845.1	1261.68	243.46	305.046	-5.2	ns
flgF (WP_006881570.1)	236.927	82.0141	54.171	-2.9	ns
flgE (WP_026027395.1)	619.483	338.421	385.431	ns	ns
flgD (WP_013856252.1)	992.065	333.312	220.056	-3.0	ns
flgC (WP_010319383.1)	5470.65	1634.97	992.253	-3.3	ns
flgB (WP_013856251.1)	4452.93	1073.12	771.889	-4.1	ns
cheR (WP_013856250.1)	2118.36	310.161	347.366	-6.8	ns
chew (WP_013856249.1)	2889.64	683.392	557.727	-4.2	ns
flgA (WP_043004140.1)	112.189	189.412	240.587	ns	ns
flgM (WP_013856247.1)	1657.65	279.441	266.407	-5.9	ns
flgN (WP_013856246.1)	849.376	226.752	245.953	-3.7	ns
flgP (WP_017043110.1)	2526.36	595.867	355.34	-4.2	ns
WP_013856244.1	401.789	80.6444	86.1174	-5.0	ns
flgT (WP_019281754.1)	339.406	148.865	107.485	-2.3	ns
VAR_RS0102305	21834.7	33357.1	22907.7	ns	ns
WP_026027396.1	1396.51	880.517	498.776	ns	ns
flaA (WP_017045944.1)	7543.89	2803.26	1071.3	-2.7	-2.6

flgL (WP_019281751.1)	356.513	192.279	107.453	ns	ns
flgK (WP_019281752.1)	182.764	87.2139	40.9505	-2.1	-2.1
flaC (WP_026029038.1)	22960.9	1829.66	1880.75	-12.5	ns
flgJ (WP_013856257.1)	63.5011	50.5542	43.3766	ns	ns
flgI (WP_019281753.1)	110.014	73.702	60.0677	ns	ns
flgH (WP_017045947.1)	95.3077	61.1727	34.6172	ns	ns
flgG (WP_010319379.1)	154.99	75.1964	53.9199	-2.1	ns
VAR_RS0102350	206.088	75.1498	50.6109	-2.7	ns
WP_019281759.1	28.3258	34.4234	44.4516	ns	ns
WP_019281760.1	13.2131	15.6022	25.9253	ns	ns
WP_019281761.1	14.7701	10.0167	9.17567	ns	ns
WP_019281762.1	173.367	51.4949	43.6861	-3.4	ns
WP_019281763.1	658.807	228.179	237.553	-2.9	ns
WP_043004148.1	284.354	126.086	114.275	-2.3	ns
WP_019281765.1	1131.32	173.517	112.271	-6.5	ns
fliL (WP_013855488.1)	473.644	129.04	99.0488	-3.7	ns
motB (WP_017044193.1)	1016.9	357.034	341.787	-2.8	ns
pomA (WP_013857399.1)	2871.64	828.47	582.023	-3.5	ns
motX (WP_013855917.1)	1576.73	596.259	907.818	-2.6	ns
motY (WP_010318471.1)	84.8421	28.7748	22.8028	-2.9	ns

[a] Only fold change values with $p < 0.05$ are shown. ns denotes that not significant differences were found.

Table S7. Expression of type VI secretion systems (T6SS) and outer membrane permeases at 25 ºC and 15 ºC and under iron deficiency. Darker colour shadows denote higher expression.

	Expression level (FPKM)			Fold change[a]	
	Fe(+) 25ºC	Fe(-) 25ºC	Fe(-) 15ºC	Fe(+) 25ºC vs Fe(-) 25ºC	Fe(-) 25ºC vs Fe(-) 15ºC
T6SS1					
vipA (WP_050934223.1)	814.443	2542.05	843.377	3.1	-3.0
vipB (WP_026027753.1)	549.576	1947.43	562.651	3.5	-3.5
lysozyme (WP_013868295.1)	175.989	951.569	255.543	5.4	-3.7
vasA (WP_019282041.1)	27.2836	87.4775	33.3469	3.2	-2.6
vasB (WP_019282042.1)	53.479	169.466	42.6442	3.2	-4.0
fha (WP_019282043.1)	76.1711	360.377	77.2212	4.7	-4.7
vasL WP_019281677.1)	27.7933	73.3933	21.8023	2.6	-3.4
vasK (WP_017045001.1)	40.6847	124.476	23.7704	3.1	-5.2
vasJ (WP_019281678.1)	98.3314	347.132	53.0565	3.5	-6.5
vasI (WP_019281679.1)	13.6323	34.1923	7.73133	ns	ns
vasH (WP_029388109.1)	67.254	188.39	35.6307	2.8	-5.3
clpB (WP_029388110.1)	48.4923	222.433	51.9803	4.6	-4.3
vasF (WP_050928076.1)	55.282	262.42	43.7926	4.7	-6.0
vasE (WP_017044997.1)	17.8958	77.1511	21.8753	4.3	-3.5
vasD (WP_019281682.1)	152.144	551.612	121.142	3.6	-4.6
T6SS2					
WP_013856698.1	3.12719	54.2805	45.8229	17.4	ns
vtsI (WP_019282546.1)	1.34988	13.0757	27.973	9.7	2.1
vtsH (WP_013856696.1)	2.857	39.8497	85.4617	13.9	2.1
vtsG (WP_019282547.1)	3.47447	51.6916	114.048	14.9	ns
vtsF (WP_019282548.1)	5.25545	86.7504	104.071	16.5	ns
vtsE (WP_029189804.1)	0.98833	26.5766	73.3223	ns	ns
vtsD (WP_019282549.1)	0.76644	28.3116	79.6949	36.9	2.8
vtsC (WP_013856691.1)	1.21379	14.9605	55.5803	ns	ns
vtsB (WP_019282550.1)	2.02319	38.3401	60.443	19.0	ns
vtsA (WP_013856689.1)	1.75907	49.4278	124.767	28.1	2.5
clpV (WP_019282551.1)	2.44775	33.3544	106.206	13.6	3.2
vasB (WP_019282552.1)	1.83427	33.0786	74.851	ns	ns
vasA (WP_017082793.1)	3.71716	23.035	88.1314	6.2	3.8
lysozyme (WP_010446924.1)	11.8391	90.4735	289.851	7.6	ns
vipB (WP_013856684.1)	6.51395	34.8136	106.012	5.3	ns
vipB (WP_004731267.1)	54.793	559.506	2403.55	10.2	4.3
vipA (WP_013856682.1)	84.2777	1021.22	3450.89	12.1	ns
hcp (WP_013856681.1)	57.3981	283.076	800.058	4.9	2.8
vasL (WP_029189803.1)	101.404	1040.56	2192.04	10.3	ns
vgrG (WP_029388295.1)	18.0072	230.97	557.787	12.8	2.4
WP_029189801.1	7.43758	66.973	137.501	ns	ns
PARR domain WP_013856676.1	3.92307	18.1195	52.0806	4.6	2.9

[a] Only fold change values with $p < 0.05$ are shown. ns denotes that not significant differences were found.

Table S8. Expression of heme uptake and siderophore systems at 25 ºC and 15 ºC and under iron deficiency. Darker colour shadows denote higher expression.

	Expression level (FPKM)			Fold change[a]	
	Fe(+) 25ºC	Fe(-) 25ºC	Fe(-) 15ºC	Fe(+) 25ºC vs Fe(-) 25ºC	Fe(-) 25ºC vs Fe(-) 15ºC
feoB (WP_017045230.1)	213.2	1255.9	589.1	5.9	-2.1
Heme uptake system				ns	ns
huvA (WP_029388256.1)	6.3	212.3	1287.1	33.9	6.1
huvZ (WP_017044790.1)	5.1	143.0	186.3	27.8	ns
huvX (WP_026027544.1)	144.4	3558.2	1592.7	24.6	-2.2
huvB (WP_019282390.1)	8.0	383.0	268.0	47.6	ns
huvC (WP_010319753.1)	24.5	145.2	85.9	5.9	ns
huvD (WP_043004479.1)	3.4	284.5	65.4	84.0	-4.4
Vanchrobactin system					
vabB (WP_019281789.1)	51.1	6875.3	4367.6	134.6	ns
vabS (WP_019281790.1)	7.0	139.8	112.7	20.0	ns
vabF (WP_019281791.1)	14.5	758.5	601.6	52.2	ns
mbtH (WP_019281792.1)	1.3	367.0	225.6	ns	ns
vabH (WP_019281793.1)	3.1	383.9	492.7	125.1	ns
fvtA (WP_019281795.1)	20.2	3511.5	1767.0	173.7	ns
vabD (WP_017045634.1)	3.8	244.3	125.9	64.8	-1.9
tonB (WP_050934211.1)	12.8	47.6	124.4	3.7	2.6
vabR (WP_029388129.1)	1.8	28.2	30.8	15.4	ns
vabG (WP_019281786.1)	11.2	1037.3	927.6	92.7	ns
vabA (WP_013857267.1)	92.7	717.7	691.5	7.7	ns
vabC (WP_043004165.1)	10.9	1940.7	2476.8	177.6	ns
vabE (WP_019281788.1)	6.4	334.7	523.4	52.4	ns
Piscibactin system					
araC1 (WP_019281874.1)	0.7	120.9	825.5	172.9	6.8
araC2 (WP_019281875.1)	0.7	12.2	47.4	16.7	3.9
frpA (WP_019281876.1)	0.9	123.2	479.0	135.1	3.9
irp8 (WP_019281877.1)	0.4	19.6	69.5	45.4	3.5
irp2 (WP_019281878.1)	0.2	25.5	110.9	125.0	4.3
irp5 (WP_019281883.1)	0.4	50.9	307.8	ns	ns
irp9 (WP_019281882.1)	0.8	84.2	529.3	99.8	ns
irp4 (WP_019281881.1)	0.8	54.0	157.0	ns	ns
irp3 (WP_019281880.1)	0.4	25.9	87.6	ns	ns
irp1 (WP_019281879.1)	0.4	42.4	187.7	97.4	4.4
frpB (WP_017046020.1)	1.5	32.1	119.9	21.6	3.7
frpC (WP_019281884.1)	1.1	15.0	69.5	13.8	4.6
Ferric siderophore reductase					
WP_017045538.1	100.998	1828.74	1981.8	18.1	ns

[a] Only fold change values with $p < 0.05$ are shown. ns denotes that not significant differences were found.

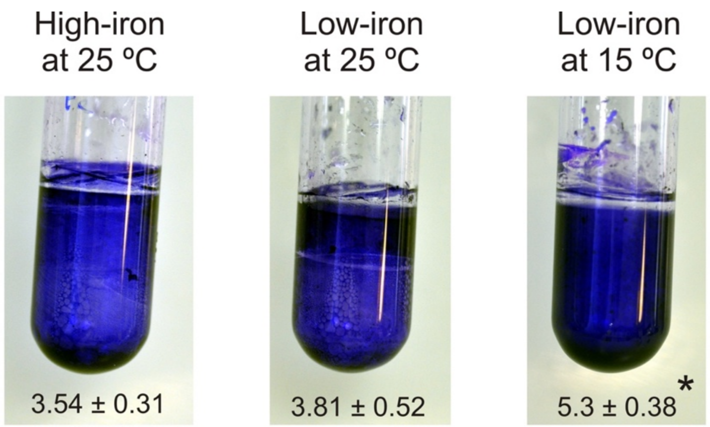

Figure S1. Biofilm formation, using the Crystal Violet method (see Material and Methods), of *V. anguillarum* RV22 at 25 ºC under high- and low-iron conditions and at 15 ºC under low iron. Numbers indicate A_{570} values with standard deviations among three replicas. Asterisk denotes statistically significant differences.

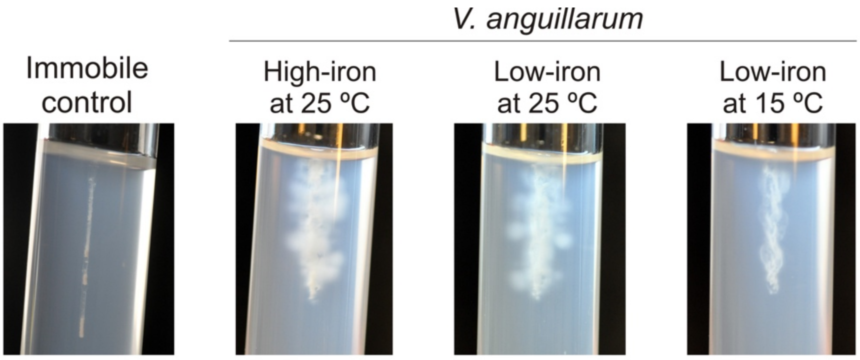

Figure S2. Motility, using the soft agar method, of *V. anguillarum* RV22 at 25 ºC under high- and low-iron conditions and at 15 ºC under low iron. *Photobacterium damselae* subsp. *piscicida* strain DI21 was used as non motile control.

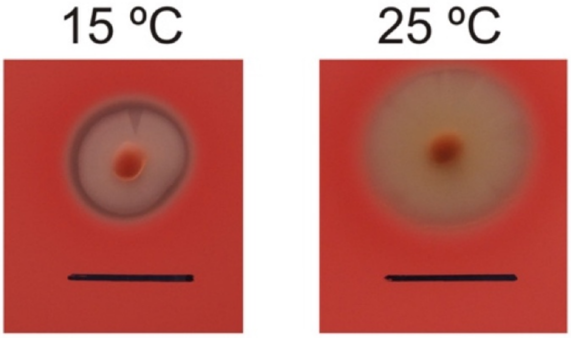

Figure S3. Haemolytic activity of *V. anguillarum* RV22 at 15 ºC and 25 ºC. A loopful of cells scratched from CM9 plates containing 2,2'-dipyridyl 50 µM and incubated at 25 ºC or 15 ºC were deposited in Columbia Agar plates and incubated for 48 h. Bar indicates 1 cm.
